# The role of microglia in processing and spreading of bioactive tau seeds in Alzheimer’s disease

**DOI:** 10.1186/s12974-018-1309-z

**Published:** 2018-09-18

**Authors:** Sarah C. Hopp, Yang Lin, Derek Oakley, Allyson D. Roe, Sarah L. DeVos, David Hanlon, Bradley T. Hyman

**Affiliations:** 1 Alzheimer’s Disease Research Laboratory, Department of Neurology, Mass General Institute for Neurodegenerative Disease, Massachusetts General Hospital and Harvard Medical School, 114 16th Street, Charlestown, MA 02129 USA; 2 Northeastern University, 360 Huntington Ave, Boston, MA 02155 USA; 3 Quanterix Corporation, 113 Hartwell Ave, Lexington, MA 02421 USA

**Keywords:** Tau, Microglia, Alzheimer’s disease

## Abstract

**Background:**

Misfolding of microtubule-associated protein tau (MAPT) within neurons into neurofibrillary tangles is an important pathological feature of Alzheimer’s disease (AD). Tau pathology correlates with cognitive decline in AD and follows a stereotypical anatomical course; several recent studies indicate that tau pathology spreads inter-neuronally via misfolded tau “seeds.” Previous research has focused on neurons as the source of these tau seeds. However, recent studies as well as the data contained herein suggest that microglia, the resident immune cells of the central nervous system, play a direct role in the spread of tau pathology.

**Methods:**

Primary adult microglia were isolated from human AD cases and the rTg4510 tauopathy mouse model and used for analysis of gene expression, tau protein by Simoa technology, and quantification of tau seeding using a highly sensitive fluorescence resonance energy transfer (FRET) biosensing cell line for tau seeding and aggregation.

**Results:**

Here, we show that microglia isolated from both human tauopathy and AD cases and the rTg4510 tauopathy mouse model stably contain tau seeds, despite not synthesizing any tau. Microglia releases these tau seeds in vitro into their conditioned media (CM). This suggests that microglia have taken up tau but are incapable of entirely neutralizing its seeding activity. Indeed, when in vitro microglia are given media containing tau seeds, they reduce (but do not eliminate) tau seeding. When microglia are treated with inflammagens such as lipopolysaccharide (LPS), interleukin-1β (IL1β), tumor necrosis factor α (TNFα), or amyloid-β, their ability to reduce tau seeding is unchanged and these factors do not induce seeding activity on their own.

**Conclusions:**

Overall, these data suggest that microglia have a complex role: they are capable of taking up and breaking down seed competent tau, but do so inefficiently and could therefore potentially play a role in the spread of tau pathology.

**Electronic supplementary material:**

The online version of this article (10.1186/s12974-018-1309-z) contains supplementary material, which is available to authorized users.

## Background

Microglia are the resident immune cells of the central nervous system (CNS) and are the primary mediators of neuroinflammation, which is an important component mechanism of neurodegeneration during Alzheimer’s disease (AD). Microglia activation correlates with cognitive decline in AD [39, 41, 42]. Similarly, misfolding of microtubule-associated protein tau (MAPT) into neurofibrillary tangles (NFTs) also correlates with cognitive deficits in AD [47]. Microglia are located near tau inclusions in various neurodegenerative diseases, including AD [2, 25, 46, 48]. NFT pathology progresses in an anatomically stereotyped course [7, 24], and microglia activation precedes tau pathology in a similarly stereotyped course [12, 26, 34, 54]. Furthermore, microglia activation seems to exacerbate tau pathological phosphorylation, aggregation, and expression in mouse and cellular models [4, 17, 30–32, 43].Tau pathology induces microglia activation, suggesting a toxic reciprocal cycle between tau pathology and neuroinflammation [37, 40, 53]. Interestingly, a recent study demonstrated that elimination of microglia can actually slow the spread of misfolded tau pathology in a mouse model of rapidly progressing tau pathology [1].

On the other hand, microglial uptake of tau leads to their own degeneration [44]. A chronic pro-inflammatory environment, such as that present in AD, leads to microglia dysfunction, which has been suggested to promote tau aggregation [8, 27]. Reduction or suppression of neuroinflammation is capable of reducing tau pathology [16, 28, 29]. However, in contrast to our data herein, previous studies have shown that microglia are capable of degrading tau most efficiently, or possibly only, when they are activated via Fc/antibody- or lipopolysaccharide- (LPS) mediated mechanisms [35, 36].

Established research clearly demonstrates a correlation among microglia, microglial activation, and tau deposition, but physiological mechanisms linking these phenotypes are uncertain. Here, we asked whether microglia participate in and enhance tau pathology or act to reduce it. To address these issues, we developed an in vitro system using microglia derived from human or mouse brains to evaluate whether microglia can take up the seed-capable form of tau that has been implicated in neuron to neuron propagation of tau in Alzheimer disease, and, if so, whether they degrade and/or secrete it back to the extracellular space. We find that both uptake and secretion occur, suggesting that microglia may play an important role in the life cycle of seed-competent tau in Alzheimer disease.

## Methods

### Animals

Tg2576 transgenic mouse embryos (and wild-type littermates) were used to generate primary neuronal cultures for experiments using amyloid-β- (Aβ) conditioned media. The Tg2576 mouse expresses the 695 aa isoform of human β-amyloid precursor protein (β APP) containing the double Swedish mutation K670N, M671L with a hamster prion protein gene promoter in B6SJL F2 [21]. Nine- to eleven-month-old rTg4510, rTg21221, and wild type (WT) littermate control mice were used as well as three-month-old C57/BL6 mice (Jackson Labs) to generate primary adult microglia cultures. The rTg4510 mouse overexpresses full-length (0N4R) human tau with the P301L frontotemporal dementia mutation [45]. The rTg21221 mouse expresses WT human tau with overexpression levels similar to the rTg4510 mouse and does not exhibit tau pathology [20]. WT littermates express the CK-tTA activator transgene but only express WT mouse tau at normal levels. Both male and female mice were used and balanced across genotypes. All experiments were performed in accordance with national and institutional guidelines. All animal experiments were approved by the Massachusetts General Hospital and McLaughlin Research Institute Institutional Animal Care and Use Committees.

### Human brain samples

Human brain tissues were collected from the frontal cortex and cerebellum of patients at the time of autopsy by the Massachusetts Alzheimer’s Disease Research Center Brain Bank. All the study subjects or their next of kin gave informed consent for the brain donation, and the Massachusetts General Hospital Institutional Review board approved the protocol. The samples were quickly dissected on ice, and microglia were isolated according to the following protocol (Brain dissociation and microglia isolation). We selected cases with a relatively shorter PMI (less than 26 h) where microglia survived until at least day in vitro (DIV) 6. Clinical diagnosis was later confirmed by a neuropathologist and received a pathological diagnosis (Table 1) using standard methods [23]. Tau neuropathology was rated by Braak stage [7] based on total tau immunostaining (DAKO A0024) and Bielchowsky’s silver stain.

**Table 1:** Human cases from which primary human microglia cultures were derived

Case	Pathology	PMI
FTD-TDP	FTD, TDP-43, gliosis, Braak I, vascular	24 h
FTD-tau1	FTD-tau, Pick’s, vascular	24 h
Control	Control, Braak I	6 h
AD1	AD, braak VI, vascular, CAA	24 h
AD2	AD, Braak VI, vascular	23 h
FTD-tau2	FTD-tau, Pick’s	24 h
DLB	DLB, Braak III	26 h
PSP	PSP	12 h

### Brain dissociation and microglia isolation and culture

Human brain samples were dissected on ice to separate the white matter from the gray matter, and then the gray matter was minced for downstream use. For mice, animals were euthanized with isoflurane and the forebrain quickly dissected from the cerebellum, hindbrain, and midbrain and the forebrain was then minced on ice. A small amount of minced brain was snap frozen and saved for later protein analysis at − 80 °C, while a small amount of minced brain for RNA analysis was added to RNAlater and stored at 4 °C. Minced fresh brain tissue was added to papain and DNase enzyme mixture provided in the Adult Brain Dissociation Kit (ABDK, Miltenyi Biotec) and mechanically minced for 30 min at 37 °C using the Miltenyi ADBK protocol on an OctoDissociator (Miltenyi Biotec). Debris and red blood cells were removed per the manufacturer’s protocol. The resulting cell pellets were incubated with anti-CD11b magnetic microbeads (Miltenyi Biotec) in a 0.5% bovine serum albumin (BSA) solution and separated via positive selection using magnetic columns (Miltenyi Biotec) per the manufacturer’s instructions. It is notable that BSA at comparable concentrations can act as a seed for Aβ and enhance toxicity to microglia [13], but we did not observe notable microglia toxicity in our cultures or observe cross-seeding of tau in Tg21221 microglia. CD11b + microglia were then either immediately frozen at − 80 °C for protein analysis after lysis with PBS containing protein inhibitor cocktail (Roche) or plated at a density of 1 × 10^5^ cells per well in 24-well plates previously coated for at least 3 h with poly-D-lysine (PDL; 50 μg/ml, Sigma) for at least 3 h. Cultures were maintained at 37 °C with 5% CO_2_ in DMEM/F12 medium supplemented with 10 ng/ml recombinant species-specific macrophage colony stimulating factor (MCSF), 2 mM Glutamax, 100 U/ml penicillin, and 100 g/ml streptomycin (microglia medium). Every 3 days, half of the media was removed and replaced with fresh media. The removed conditioned media (CM) was spun down at 1000*g* to remove cell debris and frozen at − 80 °C until later analysis.

### Primary neuronal cultures

Cerebral cortices from Tg2576 mice and wild-type littermates ages E14-E16 were dissected, minced, and washed with sterile PBS as previously described [22]. At the time of dissection, a sample of the tail was taken for DNA extraction and genotyping by polymerase chain reaction (PCR). Neurons were seeded at a density of 4 × 10^5^ cells per 35 mm PDL-treated culture dishes in neurobasal (Gibco) with 2% B27 supplement (Gibco), × 1 GlutaMax (Gibco), and penicillin (100 U/ml) and streptomycin (100 μg/ml). The cultures were maintained in the same media for 14 days at which point the media was harvested and frozen at − 80 °C until use. The concentration of Aβ was quantified by a mouse/human ELISA kit (Wako, Japan) and was determined to be 7000pMol.

### Brain protein extraction

Minced frozen brain tissue was homogenized in five volumes (wt/vol) of cold PBS containing protease inhibitor cocktail (Roche) using a Tissue Tearor homogenizer (Cole-Parmer). The homogenate was centrifuged at 3000*g* for 10 min at 4 °C (3000*g* extract), and the supernatants were collected and stored at − 80 °C for later use. Total protein was quantified by bicinchoninic acid colorimetric assay (BCA, ThermoFisher Pierce).

### Tau seeding activity assay

The presence of tau seeds can be assessed with a fluorescence resonance energy transfer (FRET) -based biosensor cell line, composed of HEK293 cells that stably express the tau repeat domain (TauRD) conjugated to cerulean fluorescent protein (CFP) or yellow fluorescent protein (YFP) [19]. CFP to YFP FRET at 535 nm is measurable within these cells when there is tau aggregation, which can be induced with the application of tau aggregates or “seeds” [11, 15, 50, 51]. For the experiments herein, cells were plated at 20,000 cells per well in 96-well plates previously coated with PDL. The next day, samples were added to the plates. For microglia CM, CM was thawed and then diluted 1:1 in Opti-MEM medium (#11058-021, Life Technologies) to a final concentration of 1% Lipofectamine 2000 (#11668019, Life Technologies). Seventy-two hours later, cells were detached with trypsin, centrifuged at 300*g*, fixed with 2% paraformaldehyde (PFA) in suspension for 10 min, centrifuged at 300*g*, and resuspended in phosphate-buffered saline (PBS) for analysis by flow cytometry. For mouse brain or cell lysate, lysate was diluted in 1% Lipofectamine in Opti-MEM to 0.5 μg for brain lysate or 1 μg for cell lysate of total protein per well and cells were fixed in suspension as described above for flow cytometric analysis 12 or 36 h later, respectively. Live-cell images were captured with an EVOS cell imaging system (Thermo Fisher Scientific) immediately prior to detachment and fixation described above. Imaging was completed using excitation via a 488-nm light-emitting diode (LED), and fluorescence was acquired with a 500–550 nm FRET cube filter. After fixation, cells were pelleted and resuspended in phosphate-buffered saline (PBS) and analyzed on a flow cytometer MACSQuant VYB (Miltenyi Biotec) with excitation via a 488-nm laser and emission captured by a 525/50 nm band pass filter. Gates were drawn to select live cells and subsequently single cells by forward and side scatter channels. Twenty thousand events from the cells➔singlets gate were collected for analysis. FRET density was calculated by multiplying the percentage of FRET-positive events by the median fluorescence intensity of the FRET-positive events to generate an integrated FRET density (IFD) which was then normalized to control biosensor cells treated only with Lipofectamine. Each flow condition was performed at least in triplicate.

### Immunoprecipitation for seeding assay

To confirm that tau is driving the activity observed in the seeding assay, we used immunoprecipitation (IP) to deplete tau from brain and microglia protein lysate samples. The mouse monoclonal antibody HT7 (ThermoFisher MN-1000) was used to capture tau. Mouse IgG1 isotype-control antibody (ThermoFisher MA5–18095) or Protein G Dynabeads without antibody were used as controls. Magnetic Dynabeads were resuspended by pipetting. Twenty-five microliters of Dynabeads (750 μg) were transferred to an Eppendorf tube and placed on a magnet to remove supernatant. Dynabeads were rinsed with 100 μl PBS, then resuspended in 100 μl PBS with antibodies diluted to 0.5 μg/ml. Antibody-bead complexes were incubated on a tube rotator for 10 min at room temperature, then placed on a magnet and rinsed with 100 μl PBS. One hundred microliters of 0.5 mg/ml-cleared 3000*g* protein lysate was incubated with the beads for 10 min on a tube rotator. After 10 min, the supernatant was removed and filtered with a 0.22 μm syringe filter to sterilize the sample. Sterilized IP supernatant samples were added at 0.5 μg or 1 μg per well (based on pre-IP protein content) to the seeding assay.

### Gene expression analysis

Quantitative real-time PCR (qPCR) was performed on whole brain as well as cell suspensions of microglia collected immediately upon isolation. Samples were lysed in RLT buffer from the RNeasy Mini Kit (Qiagen), and RNA was extracted per the manufacturer’s protocol. cDNA was generated from RNA using the High-Capacity cDNA Reverse Transcription Kit (Applied Biosystems). Resulting cDNA was added at 500 ng per well to custom TaqMan Express Array qPCR plates containing dried-down primers and probes consisting of FAM (5′) Minor Groove Binder (3′) and Non-Fluorescent Quencher (3′). Primers included glyceraldehyde 3-phosphate dehydrogenase (GAPDH) as a housekeeping gene, platelet endothelial cell adhesion molecule (PECAM), CD11b, CX3CR1, myelin oligodendrocyte glycoprotein (MOG), microtubule-associated protein tau (MAPT), microtubule-associated protein 2 (MAP2), and aldehyde dehydrogenase 1 family member L1 (Aldh1l1). TaqMan Fast Universal PCR Master Mix (Applied Biosystems) was used and diluted with ultrapure H_2_O per the manufacturer’s instructions. qPCR was performed on a CFX96 thermocycler (Bio-Rad) according to Applied Biosystems cycling conditions. Melt curves were generated to rule out primer-dimer nonspecific binding. Data was analyzed by the delta-delta Ct method.

### Simoa Human Total Tau immunoassay

The concentration of human tau was determined by the Simoa Human Total Tau assay (Quanterix) by the manufacturer. Simoa digital immunoassay was chosen due to its ability to measure tau in miniscule quantities [5], and we had found traditional enzyme-linked immunosorbent assay (ELISA) was unable to quantify the amount of tau in microglia CM (data not shown). Samples were diluted to a consistent concentration of 0.002 mg/ml total protein as determined by BCA (Thermo Fisher Scientific) for the following sample types: mouse brain lysate, non-microglia cell lysate, microglia fresh cell lysate, microglia DIV3, microglia DIV6, and microglia DIV9; however, the isolation procedure per the manufacturer’s instructions for microglia includes addition of BSA such that cellular protein content was difficult to quantify accurately; as such, data is expressed as fold-change compared to WT controls. Microglia-conditioned media from DIV3, DIV6, and DIV9 were undiluted prior to the assay.

### Immunocytochemistry

Microglia were first washed five times with PBS, fixed with 2% PFA for 10 min, permeabilized with 0.1% Triton X-100 in PBS (PBS-T) for 20 min followed by blocking in 1.5% normal goat serum in PBS-T for 1 h, and incubated overnight at 4 °C with mouse anti-human tau antibody (HT7, ThermoFisher MN-1000, 1:1000) to detect rTg4510 lysate-containing human tau and/or rabbit anti-Iba1 (WAKO, 1:500) to label microglia. The next day, samples were rinsed and incubated with secondary antibodies of goat anti-mouse and goat anti-rabbit AlexaFluor 488 and 555, respectively (Invitrogen). Nuclei were counterstained with DAPI. Images were captured with an EVOS cell imaging system (Thermo Fisher Scientific) or a Zeiss Axio Imager Z epifluorescence microscope (Zeiss) with a × 20 objective.

### Microglia and inflammagen seeding experiment

**Fig. 1:**
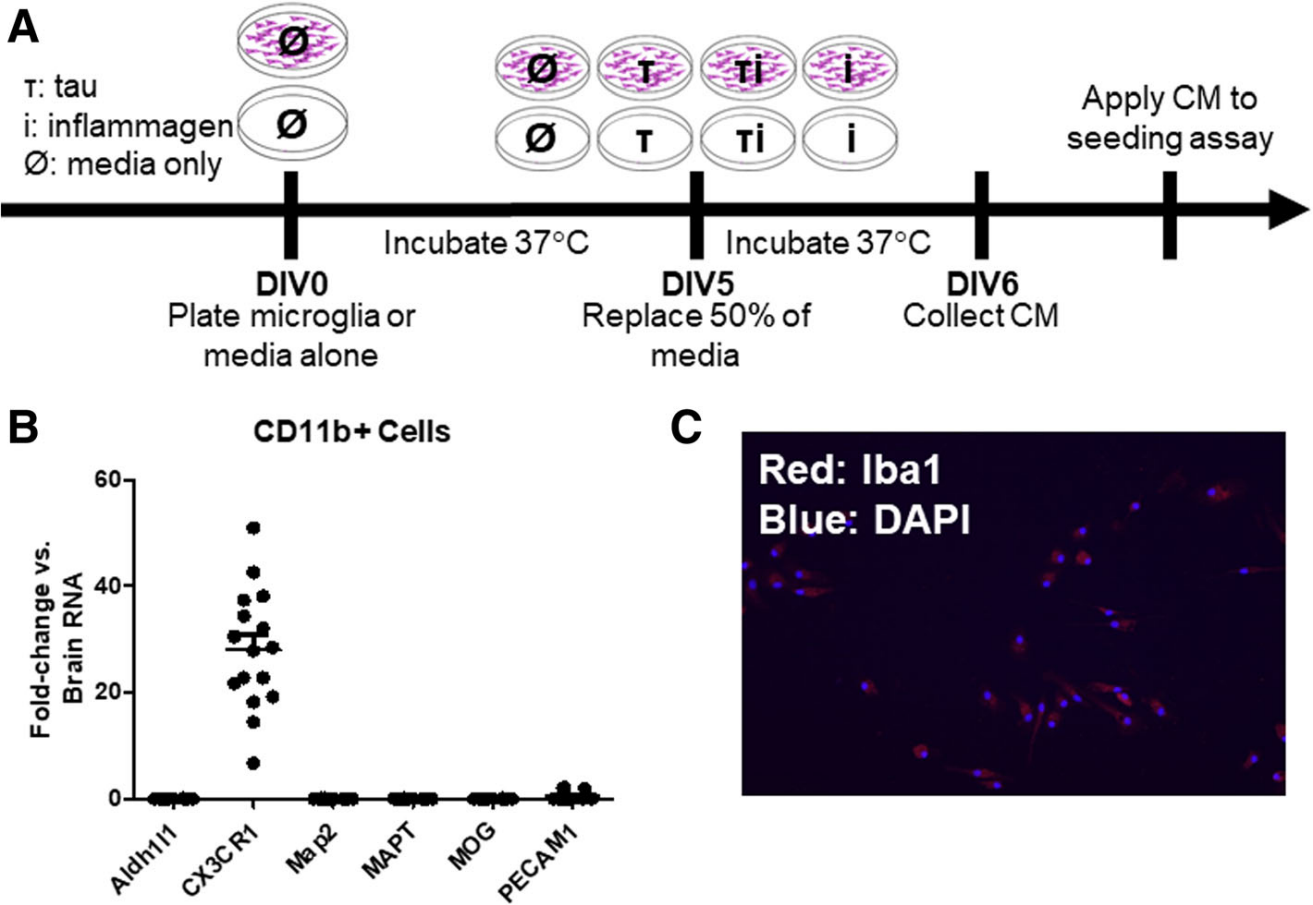
**a** Experimental design to examine the role of inflammagens on tau seeding activity. Microglia or media alone was plated at DIV0. At DIV5, half of the media was replaced with fresh media containing rTg4510 lysate containing misfolded tau seeds (τ) inflammagen of varying types (i), or media only (Ø). Twenty-four hours later, media was collected for later application to the seeding assay. **b** Gene expression of various cell type-specific transcripts was quantified by qPCR. Isolated microglia are enriched for the microglia marker CX3CR1 but not markers for astrocytes (Aldh1l1), neurons (Map2, MAPT), oligodendrocytes (MOG), or endothelial cells (PECAM1). Importantly, isolated microglia do not express the gene for tau, MAPT. **c** Representative fluorescent photomicrograph of Iba1 (red) and DAPI nuclear counterstain (blue) showing typical density and purity of mouse microglia cultures at × 20

For experiments examining the effects of inflammagens on seeding, microglia from 3-month-old C57BL/6J mice were used and seeded at 1 × 10^5^ cells/well in PDL-coated 24-well plates. For control, microglia media were added to empty PDL-coated wells. See Fig. 1a for an illustration of the experimental design. On DIV5, half of the media from all the wells was replaced with a 50/50 mix of primary neuron media (see above) and microglia media (see above). This replacement media contained various inflammagens, including 3.5 nM Aβ (Tg2576 primary neuron-conditioned media), 0.15 nM Aβ (WT primary neuron-conditioned media), 10 ng/ml lipopolysaccharide (LPS, Sigma), 10 ng/ml recombinant TNFα (R&D Systems), or 10 ng/ml IL-1β (R&D Systems). Tg4510 total brain lysate was added at a final concentration of 5 μg total protein per well. Microglia or media-only wells were incubated for 24 h at 37 °C, and after 24 h, their media were collected for application to the seeding assay. Samples without microglia were utilized as a control to test for non-cell mediated-protein degradation of tau and were treated the same as wells containing microglia by incubation at 37 °C for 24 h. Microglia treated with rTg4510 lysate were fixed in 2% PFA and labeled as described above for Iba1 (WAKO, rabbit) and human tau (HT7, Thermo, mouse) to confirm uptake of tau. An aliquot of microglia CM was saved for analysis of cytotoxicity by measurement of lactate dehydrogenase (LDH) by LDH-Cytotoxicity Assay Kit II (Abcam).

### Statistical analysis

Data was analyzed using GraphPad Prism 5. All data were expressed as mean ± standard error of the mean (SEM). Two-group comparisons were performed by unpaired two-tailed *t* test, unless stated otherwise. Comparison among three or more groups was performed by analysis of variance (ANOVA) with a Bonferonni post-hoc for multiple comparisons, unless stated otherwise. *p* values < 0.05 were considered significant. Unless otherwise indicated, each data point represents averaged replicates from one mouse.

## Results

### Isolated microglia are relatively pure and do not express the tau gene

In order to determine purity of cells isolated by CD11b magnetic microbead isolation, we performed qPCR on isolated microglia from pooled WT and rTg4510 microglia (Fig. 1b). The microglia-specific gene transcript for CX3CR1 was significantly enriched ~ 28-fold (*p* < 0.0001) compared to whole brain gene expression. There were nearly undetectable levels of Aldh1l1 (astrocytes), Map2 (neurons), or MOG (oligodendrocytes) and very low levels of PECAM (endothelial cells) compared to CX3CR1. Importantly, the lack of MAPT gene transcription by microglia indicate that microglia are not producing tau protein themselves. There was minor contamination of endothelial cells as noted by the remaining presence of PECAM. We visually confirmed purity of the cultures with immunocytochemistry of the microglia marker Iba1 with a DAPI nuclear counterstain (representative image in Fig. 1c).

### rTg4510 adult primary microglia do not express tau mRNA but do contain tau protein and release it

**Fig. 2:**
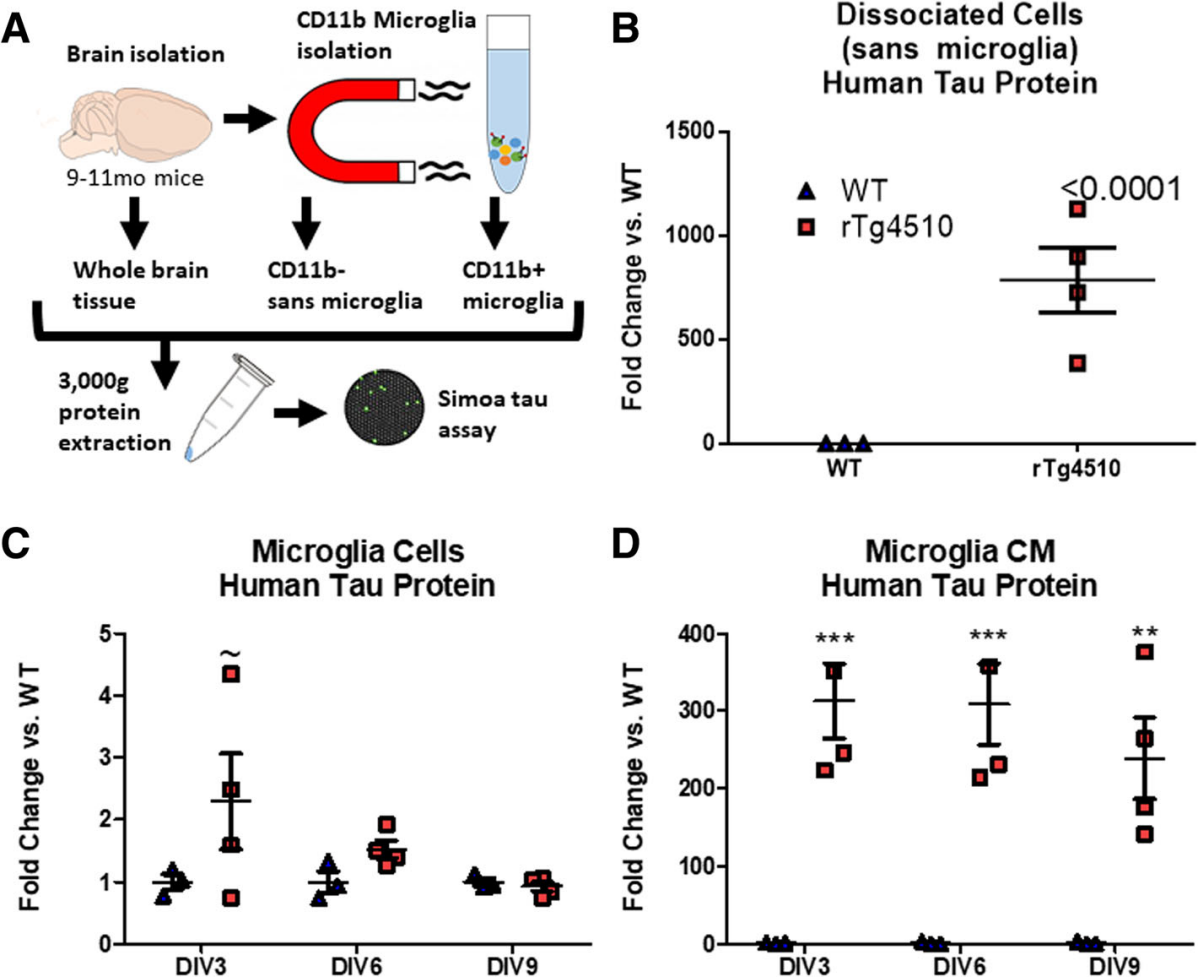
Human tau was quantified by Simoa (single molecule array) assay and is expressed here as fold-change from WTs. **a** Experimental design. **b** Lysate from dissociated cells depleted of microglia from rTg4510 mice contain high levels of human tau, significantly more than WT littermates (*p* < 0.001). **c** Microglia isolated from rTg4510 mice contain human tau protein on DIV3 by two-tailed *t* test (*p* = 0.03) even though they do not express the human tau gene MAPT (Fig. 1). This expression tapers off over time (*p* < 0.05 on DIV6 and DIV9). However, a two-way ANOVA did not reveal any main effects of genotype or timepoint. **c** Conditioned media from rTg4510 microglia contain significantly more tau than conditioned media from WT littermates on DIV3 and DIV6 (*p* < 0.001) and on DIV9 (*p* < 0.01) as measured by a two-way ANOVA with a Bonferroni post-hoc test. *** *p* < 0.001; ** *p* < 0.05, and ~*p* < 0.05 by *t* test at the given timepoint but not by ANOVA

As examined above, we found that microglia do not express the tau gene MAPT (Fig. 1), which is in line with previous research on cell-type specificity of tau gene expression [3, 55]. We therefore set out to examine whether tau protein could be found in microglia from the rTg4510 tauopathy mouse model and whether the human tau within those microglia was capable of inducing seeding. Mice were sacrificed at 9–10 months of age, an age at which point tangles are present in the rTg4510s as well as cortical cell loss [45]. Microglia from rTg4510 mice and WT littermates (*n* = 3/genotype) were isolated and cultured for 9 days. Every 3 days, one well of microglia and their conditioned media from each mouse were collected and frozen for later analysis by Quanterix Simoa protein assay for human tau (Fig. 2a). The isolation procedure for microglia includes addition of BSA, so data is expressed as fold-change compared to WT littermates. This also controls for the background of the assay as WT mice should not express human tau, but the assay showed very small amounts of human tau in WT mice. As a positive control for tau expression, non-microglia cells (neurons, astrocytes, etc.) were collected from each animal. Non-microglia cells from rTg4510 brains expressed large amounts of human tau protein, as expected (Fig. 2b), significantly more than WT littermates as measured by two-tailed *t* test (*p* < 0.0001). Interestingly, microglia isolated from rTg4510 mice also showed minute quantities of human tau protein expression on DIV3 (*p* = 0.03 by two-tailed *t* test) which tapered off over time in vitro (*p* > 0.05 on DIV6 and DIV9). This may suggest that microglia contain tau despite not expressing MAPT gene transcripts (Fig. 2c), but also suggesting that they are degrading or releasing it over time, since the amount tapers off, although a two-way ANOVA did not show a main effect of either genotype or timepoint on tau protein expression. We then examined microglia-conditioned media (CM) from rTg4510 mice and found that their media contained significantly more human tau protein compared to WT microglia on DIV3 and DIV6 (*p* < 0.001) and DIV9 (*p* < 0.01) as quantified by two-way ANOVA with a Bonferroni post-hoc test Fig. 2d), which also tapered off over time in vitro, demonstrating that microglia releases tau in vitro but do not generate more during that time.

### rTg4510 adult primary microglia contain and release bioactive tau seeds

We then determined if the tau within microglia and in their conditioned media contained bioactive tau seeds. The presence of tau seeds can be assessed by a fluorescence resonance energy transfer (FRET) -based biosensor cell line, composed of HEK293 cells that express the tau repeat domain (TauRD) conjugated to CFP or YFP [19]. CFP to YFP FRET at 535 nm is measurable within these cells when tau seeds are applied to the assay [11, 15, 50, 51]. Here, in order to control for the over-expression of mutant tau in the rTg4510 mice, we also used rTg21221 mice which overexpress the same amount of wild-type human tau, as well as WT littermates. A 3000-*g* lysate was generated from whole brain homogenate, microglia-depleted cell suspensions (sans microglia), and microglia-enriched cell suspensions.

First, to confirm the function of the assay, 0.5 μg of total protein per well of whole brain lysate was applied to the HEK-TauRD-CFP/YFP cells (Additional file 1: Figure S1A) with 1% Lipofectamine (to promote internalization) and cells were fixed for flow cytometry 12 h later. rTg4510 whole brain lysate induced significant FRET seeding activity compared to rTg21221 or WT lysate (*p* < 0.001 by one-way ANOVA), which were not significantly different from each other and displayed no seeding activity (Additional file 1: Figure S1B-H).

Similarly, microglia-depleted and microglia-enriched cell-suspension lysates were applied at 5 μg total protein per well to the HEK-TauRD-CFP/YFP cells with Lipofectamine and fixed for flow cytometry 36 h later. The quantity of protein was higher and time-adjusted for the cell lysates to account for inaccurate protein quantification by BCA protein assay due to bovine serum albumin (BSA) used during cell isolation. Microglia-depleted (sans MG) samples (Additional file 2: Figure S2A) were used as a control to make sure that the process of isolating cells did not itself effect the seeding assay using the CD11b-negative population of cell isolated by magnetic microbeads. Dissociated cells depleted of microglia from rTg4510 mice induced significant FRET seeding activity compared to rTg21221 or WT microglia-depleted cell lysates (*p* < 0.001 by one-way ANOVA), which were not significantly different from each other and displayed no seeding activity (Additional file 2: Figure S2B-H). Interestingly, it appears that the normalized IFD was up to 10-fold higher for the dissociated cells compared to total brain homogenate, and this seems to be due to a higher fluorescence intensity of the dissociated cell lysate (Additional file 2: Figure S2F) compared to total brain lysate (Additional file 1: Figure S1F) even though the percentage of FRET-positive cells is not higher.

To further confirm the validity of the seeding assay, we immunodepleted tau from rTg4510 or WT brain lysate by IP (*n* = 3) with the anti-human tau antibody HT7 or a control IgG (Additional file 3: Figure S3A). IP beads alone were used as an additional control. A two-way ANOVA with Bonferroni post-test revealed that IP with HT7 significantly reduced seeding activity (~ 75% reduction) in rTg4510 brain lysate compared to lysate that did not undergo IP, while IP beads alone and IgG had no effect (Additional file 3: Figure S3B).

**Fig. 3:**
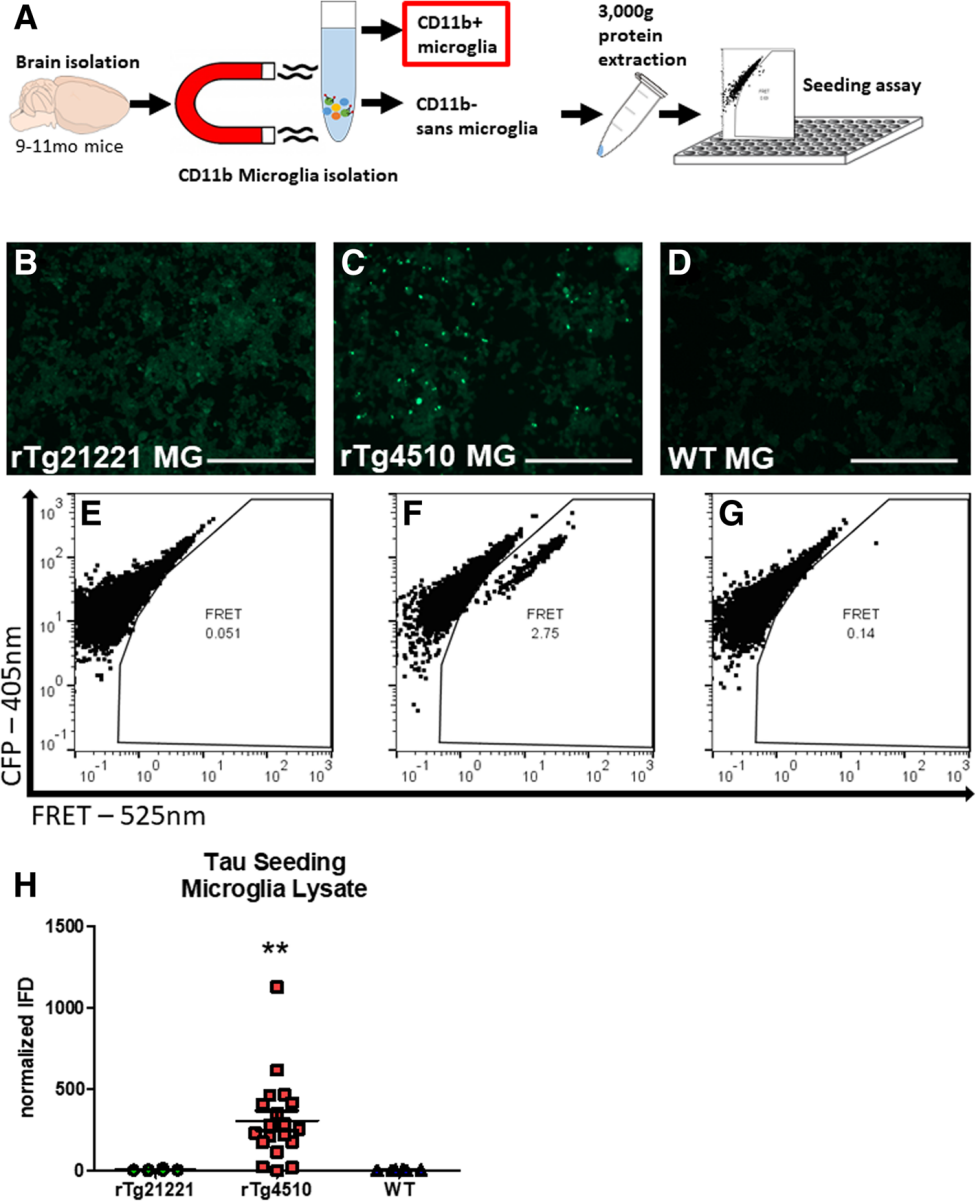
Tau seeds in microglia protein lysate were quantified using a FRET-based biosensor cell line that detects tau aggregates. **a** Experimental design. **b**–**d** Representative photomicrographs of biosensor cell line 36 h after application of rTg21221, rTg4510, and WT microglia lysate. Bright puncta visible in the rTg4510 sample in (**c**) are indicative of tau aggregates. **e**–**g** Representative flow cytometry plots for measurement of FRET seeding in (**e**) rTg21221, (**f**) rTg4510, and (**g**) WT microglia. **h** Quantification of FRET seeding by normalized (to untreated wells) integrated FRET density (IFD) and analysis by one-way ANOVA revealed that there was a significant main effect of genotype (*p* = 0.0003); Bonferroni post-test demonstrated that there is significantly more seeding in the rTg4510 microglia compared to rTg21221 or WT microglia (*p* < 0.01)

When we quantified the seeding activity of the microglia protein lysate applied at 5 μg total protein per well, (Fig. 3), a one-way ANOVA revealed a significant main effect of genotype (*p* = 0.0003) and a Bonferroni post-test demonstrated that the rTg4510 microglia lysate induced significant seeding compared to rTg21221 or WT microglia cell lysates (*p* < 0.01), which were not significantly different from each other and displayed no seeding activity (Fig. 3f). This suggests that the tau that we observed in small quantities within microglia (Fig. 2) is bioactive in that it is capable of inducing seeding in recipient cells. It is notable that while the seeding induced by rTg4510 microglia lysate is significantly higher than WT or rTg21221 controls, it is about 10-fold lower than seeding induced by non-microglia rTg4510 cell lysate in Additional file 2: Figure S2 (308.4 ± 57.6 vs. 3570 ± 752 mean ± SEM).

**Fig. 4:**
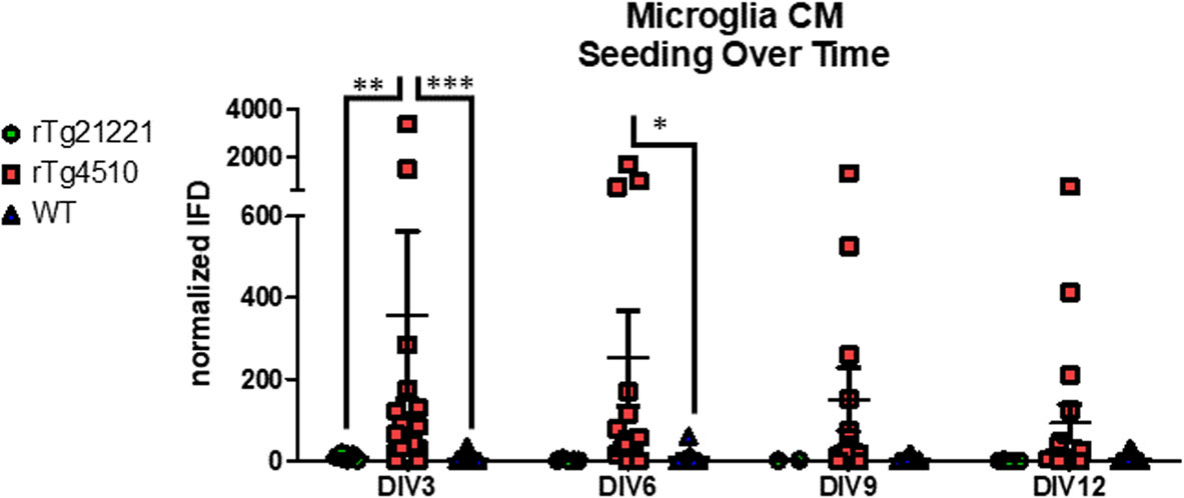
Tau seeds released by microglia in their condition media were quantified by a FRET-based biosensor cell line that detects tau aggregates. rTg4510, rTg21221, and WT microglia-conditioned media was collected on DIV3, DIV6, DIV9, and DIV12, applied to the seeding assay, and quantified by flow cytometry after 72 h. Analysis by two-way ANOVA demonstrated a significant main effect of genotype (*p* < 0.0001) but not DIV (*p* = 0.3674). Bonferonni post-test revealed that the rTg4510 microglia CM induced significantly more seeding than the rTg21221 or WT microglia CM on DIV3 (*p* < 0.01 and *p* < 0.001, respectively) and more than the WT microglia on DIV6 (*p* < 0.05). While there was still some FRET seeding activity on DIV9 and DIV12, it was not significantly higher than either the rTg21221 or WT microglia CM (Fig. 4)

Microglia, despite not expressing tau at the transcriptional level, contain seed-capable tau aggregates. We next determined if microglia releases these tau seeds in an active form, or if we were simply catching microglia in the process of degrading tau seeds. Microglia were cultured for 12 days, and every 3 days, half of the media was replaced with fresh media on DIV 3, 6, 9, and 12, and the other half of CM was used to assess seeding activity. Microglia CM was mixed in equal amounts with Optimem with a final concentration of 1% Lipofectamine. This diluted microglia CM was applied to the HEK-TauRD-CFP/YFP cells for 72 h at which point the cells were fixed in suspension and analyzed via flow cytometry (Fig. 4). CM from rTg4510 microglia induced significant seeding (Fig. 4a), while rTg21221 or WT mice did not induce any seeding activity at any DIV (Fig. 4b,c). A two-way ANOVA revealed a significant main effect of genotype (*p* < 0.0001) but not DIV (*p* = 0.3674); a Bonferonni post-test revealed that the rTg4510 microglia CM induced significantly more seeding than the rTg21221 or WT microglia CM on DIV3 (*p* < 0.01 and *p* < 0.001, respectively) and more than the WT microglia on DIV6 (*p* < 0.05). While there was still some FRET seeding activity on DIV9 and DIV12, it was not significantly higher than either the rTg21221 or WT microglia CM (Fig. 4). This is longer than the previous reported half-lives for tau in vitro ranging from 3to 20 h [9, 49].

We also immunodepleted tau from rTg4510 or WT microglia lysate (*n* = 3) by IP with the anti-human tau antibody HT7 or a control IgG (Additional file 3: Figure S3C) to test whether tau seeding from microglia lysate was due to tau or other factors. IP with HT7 did not significantly reduce tau seeding activity compared to no IP, IP without antibody (negative control), or IgG (negative control). However, the no IP, IP without antibody, and IgG rTg4510 microglia lysate samples induced significantly more seeding than their treatment-matched WT samples (*p* < 0.01, *p* < 0.05, and *p* < 0.001, respectively) while HT7-treated rTg4510 lysate was not significantly different than HT7-treated WT samples (*p* < 0.05), suggesting a partial reduction by HT7 depletion. This suggests that other factors found in microglia may influence seeding activity, such as inflammatory cytokines [18], that microglia-internalized tau may not be conformationally compatible with HT7, or the low amount of seeding seen in this set of microglia lysate experiments may be too low to achieve robust reductions.

### Human postmortem microglia also release bioactive tau seeds

**Fig. 5:**
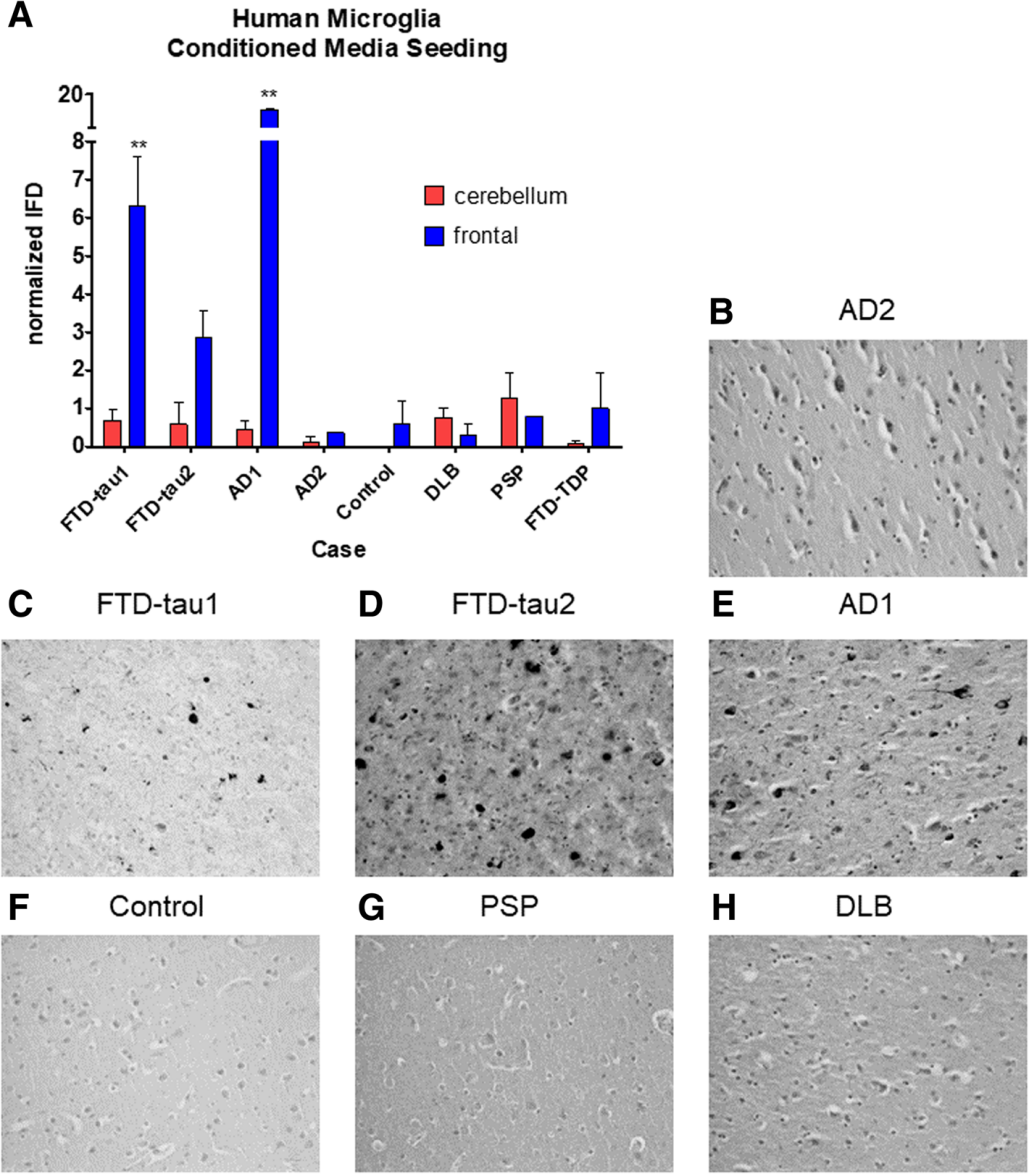
Human microglia cells from AD and tauopathy cases also release seed-competent tau into their CM. **a** Three technical replicates of microglia were cultured for conditioned media from each case and tested on a FRET-based biosensor cell line for detection of tau aggregates. A two-way ANOVA revealed a significant main effect of region (*p* = 0.0002), case (*p* < 0.0001), and an interaction between the two (*p* < 0.0001).Two cases with extensive tau pathology by neuropathological diagnoses yielded microglia CM that triggered seeding activity. Cases FTD-tau1 (frontotemporal dementia, tau variant) and AD1 (AD Braak VI) had significantly more seeding in frontal cortex microglia-conditioned media compared to cerebellum microglia-conditioned media (Bonferroni post-test, *p* < 0.001). **b**–**d** Total tau (DAKO A0024) labeled neuropathology slides of the superior frontal gyrus from the cases used to generate microglia. The superior frontal gyrus from the AD2 case (**b**) contained very little tau pathology characterized by flame-like interneuronal neurofibrillary tangles compared to cases where microglia CM did induce seeding (Fig. 5c–e) and was more similar to causes with minimal tau pathology (Fig. 5f–h)

In order to confirm our observation in a mouse model of tauopathy in human disease, we examined the release of seeds into microglia CM from cultured postmortem human cases. We first isolated and cultured microglia from human cases of AD and other neurodegenerative diseases with tau pathology. We found that conditioned media of microglia isolated from AD or FTD-tau cases were capable of inducing significantly more tau seeding than control cases in the FRET seeding assay. However, human microglia vary in yield and often die, so it was difficult to get a consistent number of live microglia or to directly compare quantitative information form case to case; here, we selected cases with a relatively shorter PMI where microglia survived until at least DIV6. Three technical replicates of microglia culture (one well each) were harvested for conditioned media. Despite these difficulties, analysis by two-way ANOVA revealed a significant main effect of region (*p* = 0.0002), case (*p* < 0.0001), and an interaction between the two (*p* < 0.0001).Two cases with extensive tau pathology by neuropathological diagnoses yielded microglia CM that triggered seeding activity. Cases FTD-tau1 (frontotemporal dementia, tau variant) and AD1 (AD Braak VI) had significantly more seeding from frontal cortex microglia CM compared to microglia isolated from the cerebellum, an area spared from tau pathology in the cases examined here (Fig. 5) as measured by two-way ANOVA and Bonferroni post-hoc (*p* < 0.01). A third case, FTD-tau2, showed a trend towards seeding in the CM from frontal cortex microglia. It was surprising that frontal cortex microglia CM from another late-stage AD case, AD2, did not show significant seeding, but upon further examination of Tau1-labeled neuropathology slides, the superior frontal gyrus from the AD2 case contained very little tau pathology characterized by flame-like interneuronal neurofibrillary tangles (Fig. 5b) compared to cases where microglia CM did induce seeding (Fig. 5c–e) and was more similar to causes with minimal tau pathology (Fig. 5f–h).

### Microglia take up and process tau seeds

**Fig. 6:**
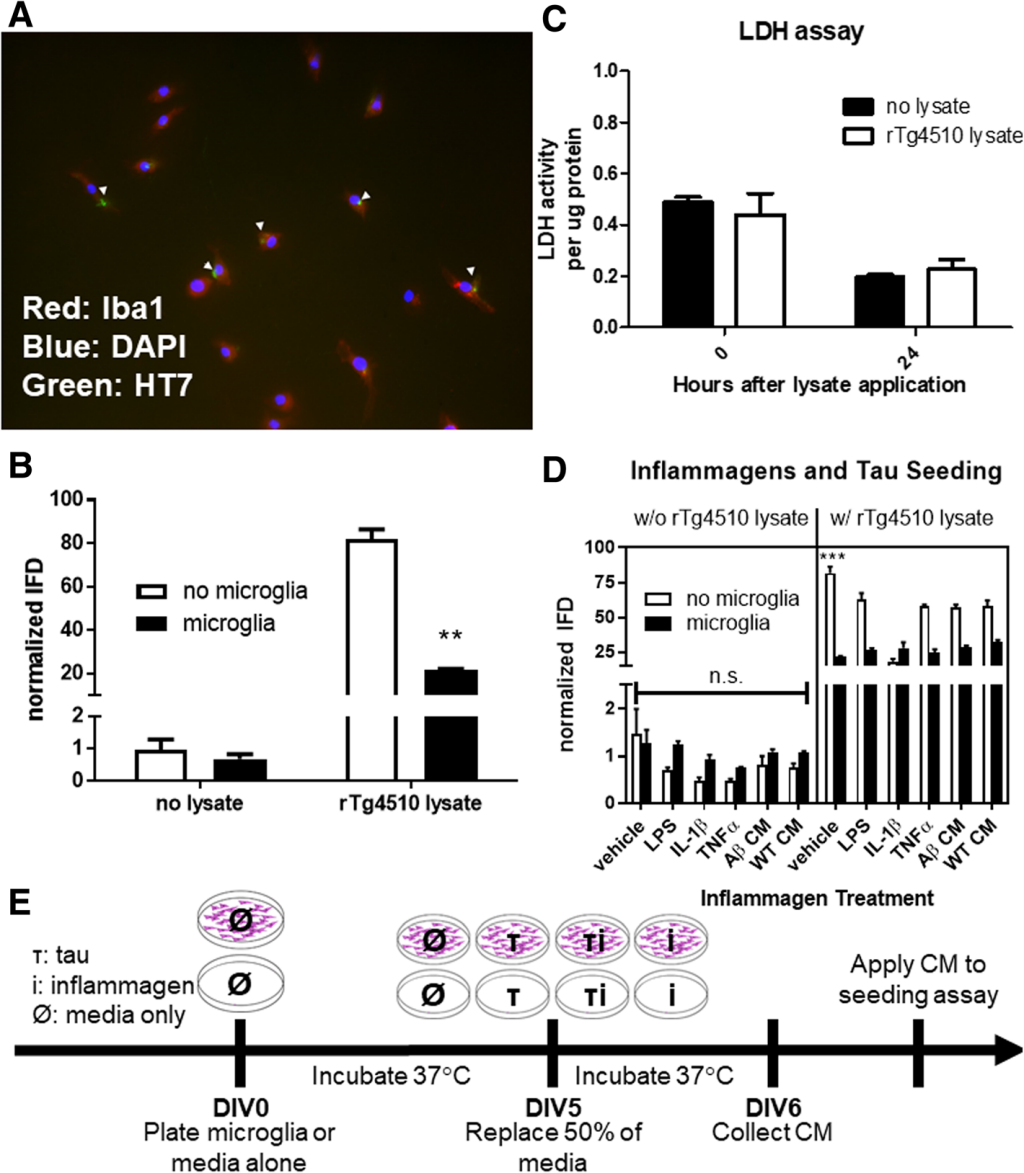
Microglia uptake and process tau, and tau is required for seeding activity independent of other inflammatory factors. **a** Wild-type microglia (labeled with Iba1, red) incubated with rTg4510 brain lysate take up tau (labeled with HT7 for human tau, green) in vitro after 24 h. **b** Microglia media or WT microglia CM on its own does not induce seeding (no lysate). However, rTg4510 lysate without microglia incubated for 24 h at 37 °C induces significantly more seeding (*p* < 0.01) than when rTg4510 lysate is incubated with microglia for 24 h at 37 °C as measured by two-way ANOVA with a Bonferroni post-hoc. **c** Tau does not have a toxic effect on microglia at the time point tested here, but there is significant toxicity with rTg4510 lysate by LDH assay at 48 h and 72 h. **d** The first panel examines whether various inflammagens are able to induce tau seeding by themselves or by triggering microglia-induced tau seeding. None of the inflammagens tested had any effect on seeding on their own (white bars) or with microglia (black bars) as measured by a two-way ANOVA with a Bonferroni post-hoc. However, there is a trend (*p* = 0.0812 and 0.0838, respectively) towards reduced IFD by IL-1β or TNFα without microglia present. The second panel examines whether there is a synergistic effect of applying these same inflammagens with rTg4510 brain lysate with or without microglia present. On its own, rTg4510 brain lysate without microglia or inflammagens induced significantly more seeding (*p* < 0.001) than any of the other inflammagens with or without microglia. Regardless of inflammagen treatment, the presence of microglia significantly reduced seeding activity and there was no difference between any of the inflammagen-treated microglia (*p* > 0.9). The presence of inflammagens did not induce microglia to increase or decrease their processing of tau, although the inflammagens by themselves seem to have an effect on the seeding assay itself. *** *p* < 0.001, ** *p* < 0.01; n.s. not significant. **e** Experimental design replicated from Fig. 1. Microglia or media alone was plated at DIV0. At DIV5, half of the media was replaced with fresh media containing rTg4510 lysate containing misfolded tau seeds (τ) inflammagen of varying types (i), or media only (Ø). Twenty-four hours later, media was collected for later application to the seeding assay

Thus far, we have shown that microglia contain minute quantities of tau protein and appear to release that tau in a form that is capable of seeding aggregates. Here, we incubated primary C57BL/6J microglia with rTg4510 lysate containing tau seeds for 24 h, harvested their conditioned media, and then fixed them for immunocytochemistry. Immunocytochemistry shows tau inclusions inside of microglia (Fig. 6a). This illustrates that microglia take up tau in vitro, which confirms previous research by other groups [36, 44]. CM from rTg4510 lysate-treated microglia was also collected after 24 h and applied to the HEK-TauRD-CFP/YFP cells. Compared to rTg4510 lysate that was diluted with media and incubated for 24 h at 37 °C in wells without microglia, lysate-media mixture that was incubated with microglia for 24 h induced significantly less seeding on the seeding assay (*p* < 0.001, Fig. 6b) as measured by two-way ANOVA with a Bonferroni post-test. This shows that microglia are, at a minimum, removing tau seeds from the media. To ensure that application of tau seeds was not causing microglia cell death, we performed an LDH assay and did not see any significant increase that might be indicative of cell death at the 24-h timepoint after application of rTg4510 lysate. We did observe toxicity at timepoints later than 24 h after application of lysate (data not shown) which represented a confounding variable for observing microglia release of tau seeds taken up in vitro.

### Microglial tau seeding is independent of Aβ and other inflammatory factors

Thus far, data from the rTg4510 mouse model and human postmortem AD and FTD cases demonstrate that microglia releases tau seeds in vitro, however, there still may be other processes by which microglia CM is inducing tau seeding, including that cytokines themselves may induce tau aggregation, which has been previously reported [18]. Indeed, we observed a weak reduction of microglia lysate tau seeding with immunodepletion with the anti-human tau antibody HT7 compared to total brain lysate immunodepletion (Additional file 3: Figure S3), suggesting that other microglia factors may influence tau seeding. To examine the possibility that other microglia factors may influence tau seeding, we added recombinant IL-1β or TNFα protein, LPS, or Aβ (in the form of Tg2576 primary-neuron CM) to the seeding assay (after a 24-h incubation at 37 °C to control for degradation of activity over time to compare to other assay conditions) to examine whether these inflammagens or other β-pleated sheet structures could induce seeding independent of tau. None of these factors induced any tau seeding on their own (Fig. 6d, first panel, white bars). To further examine whether cytokines could directly induce tau seeding, we treated primary C57BL/6J microglia with LPS, IL-1β, TNFα, or Aβ CM as in the previous seeding study in order to stimulate a more biologically relevant ensemble of inflammatory proteins from the microglia cells. The resulting microglia CM was harvested 24 h later, and none of these treatments induced seeding (Fig. 6d, first panel, black bars). Taken together, these data demonstrate that inflammatory cytokines are not sufficient to induce tau aggregation in this in vitro system. Dosages of LPS, IL-1β, and TNFα were chosen as moderate doses based on previous literature assessing microglia activation after application [14, 38].

However, inflammatory factors may still have a synergetic effect in the presence of tau seeds. When we applied rTg4510 lysate containing tau seeds in combination with the same inflammagens, we did not see any sort of synergistic effect on boosting seeding; in fact, application of inflammagens significantly reduced rTg4510-induced seeding (*p* < 0.001) across all inflammagen treatments compared to vehicle-treated no-microglia controls, as measured by a two-way ANOVA with Bonferroni post-hoc (Fig. 6d, second panel, white bars). This may be due to an effect of these factors on the seeding assay itself; as there is a slight trend towards reduced IFD without rTg4510 lysate with IL-1β (*p* = 0.0812) or TNFα (*p* = 0.0838), which could be concealed in the other groups by a floor effect (Fig. 6d, first panel).

However, when these factors are applied to C57BL/6J primary microglia in combination with rTg4510 lysate, microglia reduce the seeding activity to the same amount regardless of the presence of the inflammagen (Fig. 6d, second panel, black bars). A two-way ANOVA revealed there was no significant difference between any of the lysate-treated microglia given an inflammagen or vehicle (*p* > 0.9). This suggests that microglia are taking up tau in vitro, thus reducing the seeding activity of the resulting CM, but this is independent of their activation state with the inflammagens that we tested.

## Discussion

This study demonstrates that both human and mouse microglia contain and release tau “seeds”—that is, a form of tau that is capable of inducing tau accumulations in recipient cells [11, 15, 50, 51]. This supports a growing body of literature that microglia may play a direct role in propagation of tau pathology throughout the brain [1, 37], which has been implicated as an important aspect of the pathological timeline of Alzheimer’s disease. Previously, tau propagation has largely been thought to be due to synaptic release and possible postsynaptic uptake by “recipient” neurons [10, 33, 51, 52]. However, release of a large misfolded protein into the extracellular space might also lead to a role for microglia in clearance or processing of tau, since microglia are the obligate phagocytes of the brain. Indeed, other groups have shown that microglia are capable of taking up tau [6, 35, 36]. The data presented herein suggest that microglia may play a part in the life cycle of seed-competent tau spreading in the brain.

Tau protein and bioactive tau seeds are still present in microglia CM up to at least DIV6. This is longer than the previous reported half-life of tau in vitro which ranges from 3 to 20 h [9, 49]. This suggests that microglia releases tau seeds in vitro into their CM, indicating (1) that seed competent tau species are surprisingly long lived within microglia compared to previous in vitro half-life data; (2) that microglia are not fully processing tau into non-toxic forms; and (3) that seed-competent tau species are, surprisingly, released back to the media. Tau seeds present in microglia CM could be due to an active process, potentially via tau-containing microglial exosomes [1], or, alternatively, due to intracellular tau-mediated microglia toxicity [44] leading to release of not-yet-degraded tau seeds. We also considered that we could simply be diluting tau that is already present in the media due to media changes; if this is the case, then this suggests that normal microglial phagocytosis and degradation could be disrupted in the presence of bioactive tau seeds, as microglia are normally capable of taking up and degrading tau [6, 35, 36]. Overall, these data suggest that microglia are capable of degrading tau but not as efficiently as expected; this inefficient microglial processing of tau could open the door for microglial involvement in the spread of tau pathology in vivo.

It is clear that the progression of tau pathology and microglia-mediated neuroinflammation are reciprocally linked to each other, although the precise mechanisms by which microglia contribute to the progression of tau pathology have not been fully elucidated. Molecular weight and phosphorylation are hypothesized to influence tau propagation [51]; microglial activation has been linked to both phosphorylation and aggregation [8, 32, 37]. Microglia also directly interact with tau by taking it up and degrading it [6, 35, 36] but not without damage to themselves [44]. Despite microglial capability of degrading tau, a recent study suggested that depletion of microglia may slow the propagation of tau pathology, which may be at least in part due to microglial release of tau-containing exosomes which contributes to spreading [1]. Adoptive transfer of microglia from human tau-transgenic mice lacking the anti-inflammatory fractalkine receptor (CX3CR1) into wild-type mice induces tau pathology in recipient mice, which is reduced with anti-IL1β treatment [37], demonstrating an important role for inflammatory factors in tau pathology. We initially thought that there might be other processes by which microglia CM is inducing tau seeding, including that cytokines themselves may be inducing tau aggregation, which has been previously reported [18], but we did not observe that any of the inflammagens we tested were capable of inducing seeding on their own; of course, it cannot be ruled out that an untested combination of cytokines other than those tested would impact tau seeding and bioactivity. Further, the detection of tau seeding with HEK293 cells may not fully recapitulate tau seeding in neurons with endogenous full-length tau, which may behave differently than the tauRD used in the seeding assay here. Further, neurons express a variety of specific cytokine receptors that trigger specific intracellular signaling cascades that may confer specific vulnerability in neurons.

It is clear that the role of microglia in tau pathology is complex: the data herein suggest that microglia take up tau, fail to fully degrade it, and in turn release tau that can induce aggregation in recipient cells. In addition to containing seed-competent tau and releasing it, we demonstrate that microglia are also capable of sequestering bioactive tau and potentially degrading its seeding activity. We also showed that inflammatory molecules play a limited role in this process, which is in contrast to previous studies that demonstrate that microglia activation is required for tau uptake and breakdown by microglia [35, 36], although these studies used the BV2 microglia cell line and primary neonatal microglia, respectively, in contrast to the adult primary microglia we used for the studies herein. The role of microglia in tau propagation may be dependent on other local environment and non-cell autonomous signaling cascades not examined here.

## Conclusions

Overall, our data suggest that microglia can, at least under certain circumstances, sequester seed-competent tau species from the extracellular environment in a small sample size of human tauopathy cases and in a transgenic model of tau pathology and that the sequestered tau can be either released or broken down. Further examination of cues that regulate microglial uptake and potential degradation of misfolded tau, as well as crosstalk between microglia and neurons, may allow us to manipulate processes necessary for tau propagation and develop new treatment strategies for AD and other tauopathies.

## Additional files

Additional file 1: **Figure S1.** Confirmation of the seeding assay on brain lysate and cell lysate. A) Experimental design. B-H) Brain lysate from rTg21221, rTg4510, and WT mice was applied to the seeding assay. 12 h later, photomicrographs were taken (B-D) and cells quantified by flow cytometry (E-G). H) rTg4510 brain lysate induced significantly more seeding than rTg21221s or WTs by 1-way ANOVA (*p* < 0.001). Each point represents one mouse averaged from technically replicated triplicates. Scale bar 400 um. *** *p* < 0.001. (PNG 322 kb)

Additional file 2: **Figure S2.** Confirmation that the cell dissociation procedure did not interfere with the seeding assay functionality. A) Experimental Design. We applied microglia-depleted cell lysate (sans MG) to the seeding assay. 36 h later, photomicrographs were taken (B-D) and cells quantified by flow cytometry (E-G). H) rTg4510 cells sans MG induced significantly more seeding than rTg21221s or WTs measured by one-way ANOVA (*p* < 0.001). Each point represents one mouse averaged from technically replicated triplicates. Scale bar 400 um. *** *p* < 0.001. (PNG 306 kb)

Additional file 3: **Figure S3.** Confirmation that the seeding assay is dependent on the presence of tau using immunodepletion. A) Experimental Design. Using rTg4510 or WT mouse total brain or isolated microglia, lysate was immunodepleted and applied to the seeding assay. B) WT or rTg4510 brain lysate underwent IP with no antibody, IgG antibody control, or anti-human tau antibody HT7 and then was applied to the seeding assay. HT7 significantly (* *p* < 0.05) reduced seeding compared to the no IP control, while IP beads alone or IgG did not significantly reduce seeding. C) Microglia lysate underwent the same procedure. No IP, IP without antibody, and IgG rTg4510 samples induced significantly more seeding than treatment-matched WT controls, while HT7 treated rTg4510 samples were not significantly different from WT controls, suggesting a slight reduction in seeding with HT7 (++ *p* < 0.01, + *p* < 0.05, +++ *p* < 0.001). (PNG 83 kb)

## Abbreviations

ABDK: Adult brain dissociation kit
AD: Alzheimer’s disease
Aldh1l1: Aldehyde dehydrogenase 1 family member L1
ANOVA: Analysis of variance
Aβ: Amyloid β
BCA: Bicinchoninic acid colorimetric assay
BSA: Bovine serum albumin
CFP: Cerulean fluorescent protein
CM: Conditioned media
DIV: Day in vitro
ELISA: Enzyme-linked immunosorbent assay
FRET: Fluorescence resonance energy transfer
FTD: Frontotemporal dementia
GAPDH: Glyceraldehyde 3-phosphate dehydrogenase
IFD: Integrated FRET density
IL1β: Interleukin 1 β
LDH: Lactate dehydrogenase
LPS: Lipopolysaccharide
MAP2: Microtubule-associated protein 2
MAPT: Microtubule-associated protein tau
MCSF: Macrophage colony stimulating factor
MOG: Myelin oligodendrocyte glycoprotein
NFT: Neurofibrillary tangle
PBS: Phosphate buffer saline
PBS-T: Phosphate buffer saline with triton
PCR: Polymerase chain reaction
PDL: Poly-D lysine
PECAM: Platelet endothelial cell adhesion molecule
PFA: Paraformaldehyde
qPCR: Quantitative polymerase chain reaction
SEM: Standard error of the mean
TauRD: Tau repeat domain
TNFα: Tumor necrosis factor α
WT: Wild-type
YFP: Yellow fluorescent protein
