# Supplementary figures and images for: The role of microglia in processing and spreading of bioactive tau seeds in Alzheimer’s disease
Source: J Neuroinflammation. 2018 Sep 18;15:269. doi: 10.1186/s12974-018-1309-z (PMC6145371; doi:10.1186/s12974-018-1309-z)

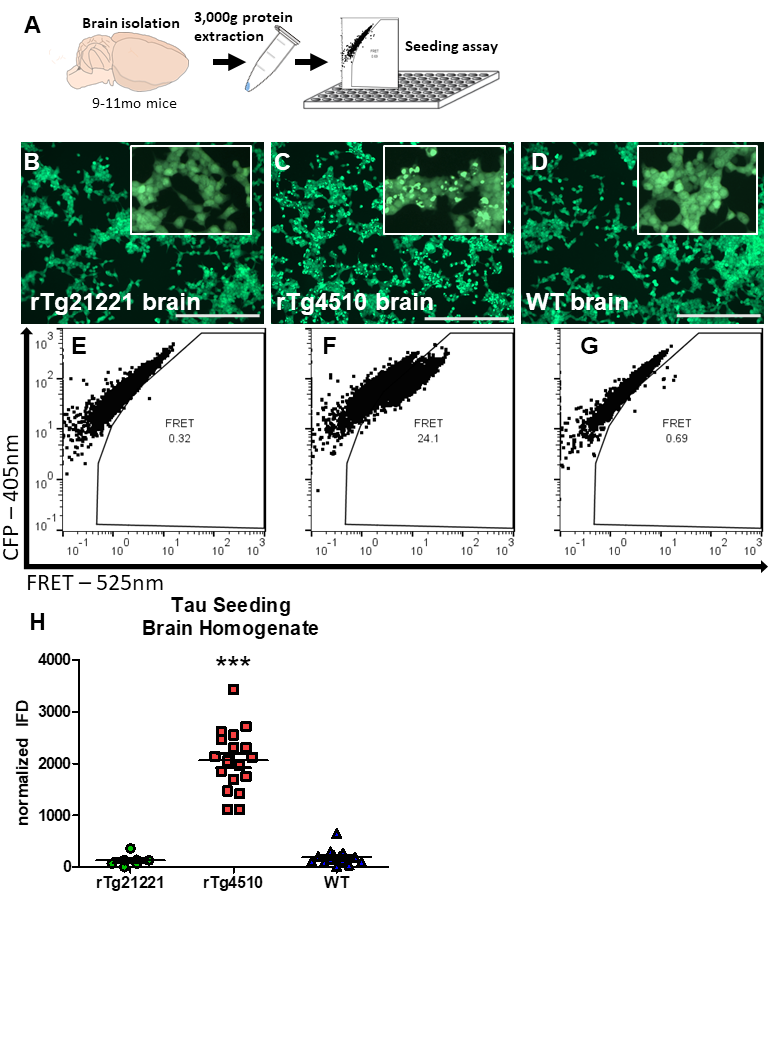

Supplement: Supplementary file 1 — Figure S1. Confirmation of the seeding assay on brain lysate and cell lysate. A) Experimental design. B-H) Brain lysate from rTg21221, rTg4510, and WT mice was applied to the seeding assay. 12 h later, photomicrographs were taken (B-D) and cells quantified by flow cytometry (E-G). H) rTg4510 brain lysate induced significantly more seeding than rTg21221s or WTs by 1-way ANOVA (p < 0.001). Each point represents one mouse averaged from technically replicated triplicates. Scale bar 400 um. *** p < 0.001. (PNG 322 kb) [file 12974_2018_1309_MOESM1_ESM.png]

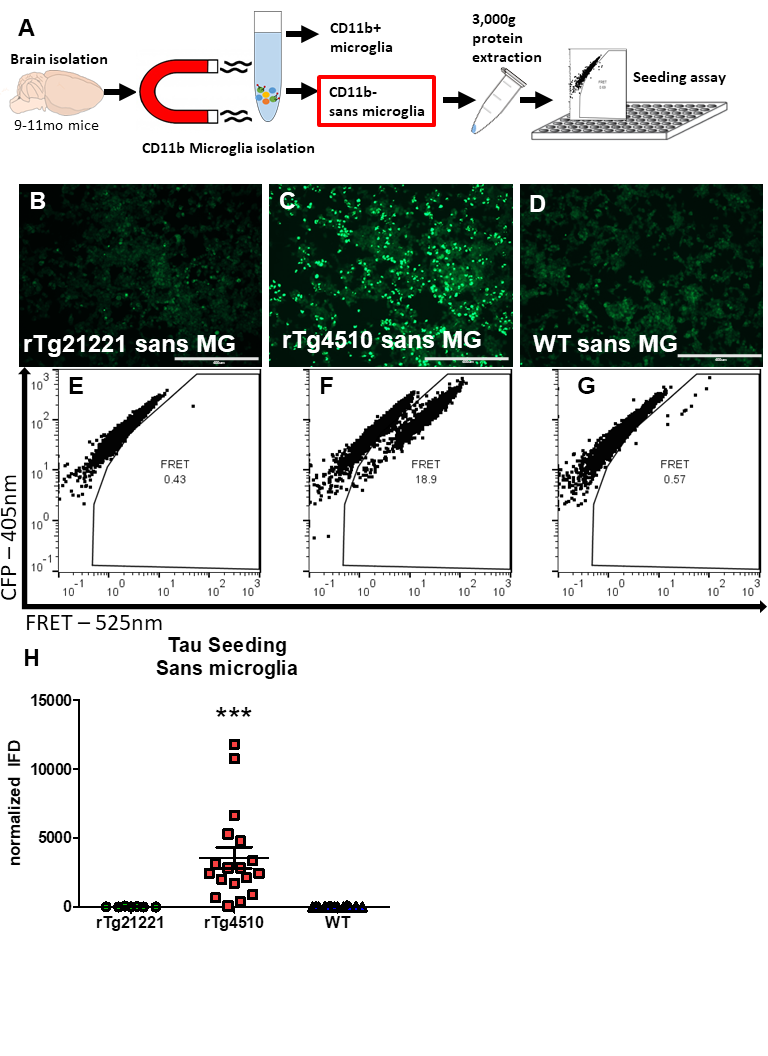

Supplement: Supplementary file 2 — Figure S2. Confirmation that the cell dissociation procedure did not interfere with the seeding assay functionality. A) Experimental Design. We applied microglia-depleted cell lysate (sans MG) to the seeding assay. 36 h later, photomicrographs were taken (B-D) and cells quantified by flow cytometry (E-G). H) rTg4510 cells sans MG induced significantly more seeding than rTg21221s or WTs measured by one-way ANOVA (p < 0.001). Each point represents one mouse averaged from technically replicated triplicates. Scale bar 400 um. *** p < 0.001. (PNG 306 kb) [file 12974_2018_1309_MOESM2_ESM.png]

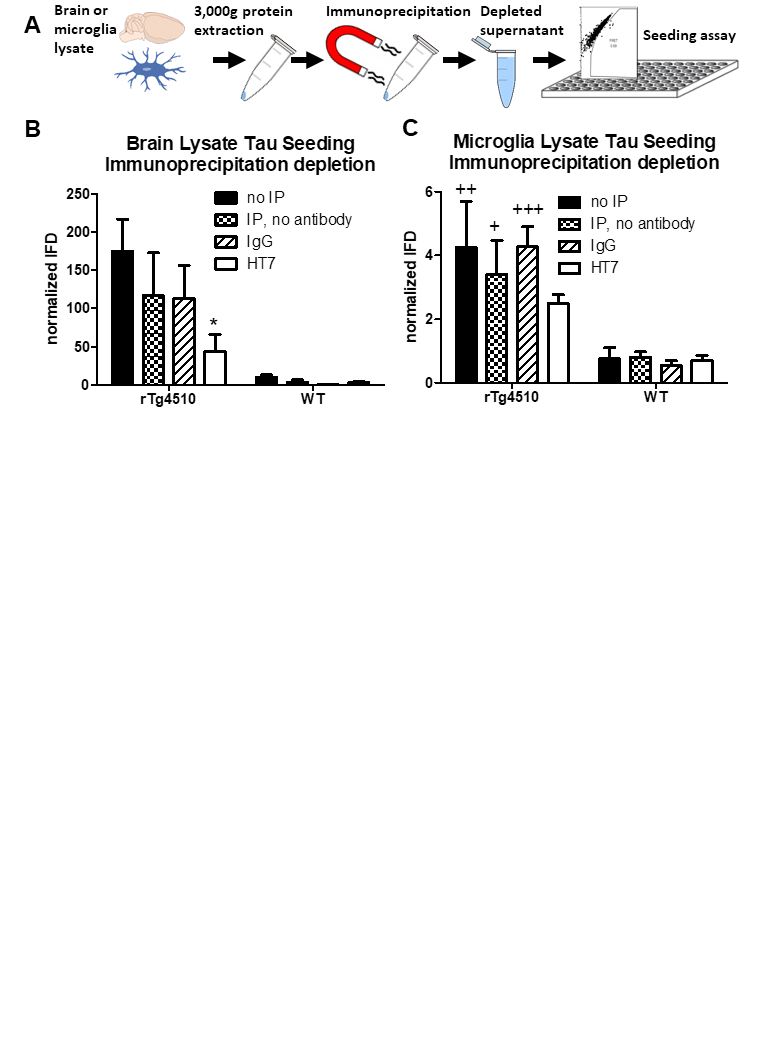

Supplement: Supplementary file 3 — Figure S3. Confirmation that the seeding assay is dependent on the presence of tau using immunodepletion. A) Experimental Design. Using rTg4510 or WT mouse total brain or isolated microglia, lysate was immunodepleted and applied to the seeding assay. B) WT or rTg4510 brain lysate underwent IP with no antibody, IgG antibody control, or anti-human tau antibody HT7 and then was applied to the seeding assay. HT7 significantly (* p < 0.05) reduced seeding compared to the no IP control, while IP beads alone or IgG did not significantly reduce seeding. C) Microglia lysate underwent the same procedure. No IP, IP without antibody, and IgG rTg4510 samples induced significantly more seeding than treatment-matched WT controls, while HT7 treated rTg4510 samples were not significantly different from WT controls, suggesting a slight reduction in seeding with HT7 (++ p < 0.01, + p < 0.05, +++ p < 0.001). (PNG 83 kb) [file 12974_2018_1309_MOESM3_ESM.png]
